# Discriminating the Effects of Prenatal Alcohol Exposure From Other Behavioral and Learning Disorders

**Published:** 2011

**Authors:** Claire D. Coles

**Keywords:** Fetal alcohol syndrome, fetal alcohol spectrum disorders, prenatal alcohol exposure, alcohol effects, neurodevelopmental effects, diagnosis, alcohol-related neurodevelopmental disorder, developmental disorders, behavioral disorders, learning disorders

## Abstract

Fetal alcohol syndrome and fetal alcohol spectrum disorders are underdiagnosed in general treatment settings. Among the factors involved in identifying the effects of prenatal alcohol exposure are (1) the evidence for prenatal alcohol exposure; (2) the effects of the postnatal, caregiving environment; (3) comorbidities; and (4) differential diagnosis, which includes identifying the neurodevelopmental effects of alcohol and discriminating these effects from those characterizing other conditions. This article reviews findings on the neurodevelopmental effects of prenatal alcohol exposure, including learning and memory, motor and sensory/motor effects, visual/spatial skills, and executive functioning and effortful control. Encouraging clinicians to discriminate the effects of prenatal alcohol exposure from other conditions may require more education and training but ultimately will improve outcomes for affected children.

Fetal alcohol syndrome (FAS) and the other conditions making up the fetal alcohol spectrum disorders (FASD) are the most common, preventable, developmental disorders in the United States and are estimated to affect as many as 1 in 100 people (Carmichael-Olson et al. 2009). However, these conditions rarely are identified in general clinical settings where children are referred for diagnosis and treatment of developmental and behavioral problems (Bertrand et al. 2005; Ryan and Ferguson 2006). This article reviews several barriers to detecting FASD and provides suggestions for discriminating the effects of alcohol from other behavioral and developmental disorders.

## Identifying Alcohol-Affected Individuals

Identifying the effects of alcohol exposure is not simple in practice. FASD is not a medical diagnosis but a description of a hypothetical spectrum of the results of exposure. Criteria exist for the identification of some of the points along this spectrum, including FAS and partial FAS (pFAS), for which there are several, not completely similar, systems recommended for clinical diagnosis (Astley and Clarren 2000; Bertrand et al. 2005; Chudley et al. 2005; Hoyme et al. 2005; Stratton et al. 1996). At the present time, there are no generally accepted “standard” recommendations for diagnosing individuals without the physical features of FAS who demonstrate only neurodevelopmental effects—that is, those people who are described by the Institute of Medicine (Stratton et al. 1996) as having alcohol-related neurodevelopmental disorder (ARND). In addition, clinicians must consider several factors in the identification of alcohol effects. These include (1) evaluation of the evidence for prenatal alcohol exposure; (2) effects of a postnatal, caregiving environment; (3) comorbidities; and (4) differential diagnosis, which includes identifying the neurodevelopmental effects of alcohol and discriminating these effects from those characterizing other conditions.

### Evidence for Prenatal Alcohol Exposure

Detecting alcohol use is challenging both during pregnancy and in retrospect. There are no biomarkers of FASD, and detecting prenatal exposure through maternal alcohol use during pregnancy can be difficult. The metabolic byproducts of alcohol are less easy to detect over time than those of many other drugs (e.g., tobacco, cocaine), and pregnancy itself affects the results of some standard measures of alcohol use (see the article by Bakhireva and Savage, pp. 56–63 in this issue). Because of these difficulties, much of the research on prenatal alcohol exposure relies on self-report data. However, alcohol use is less frequently reported than other drug use in medical records that are available to clinicians. For this reason, infants may not be accurately identified as alcohol affected. When children are older at their first referral, diagnosis relies on the self-reported recall of alcohol use after a period of years by women who may not remember well or who may have some motivation, conscious or unconscious, to downplay the extent of use. However, if the biological mother is available, her report usually is more reliable than the secondhand information and inference that can be all that the clinician has when older children present for diagnosis. Because many children have come into the care of other biological families, the foster care system, or have been adopted, exposure information often has to be obtained from medical or social service records or by hearsay from other individuals. This problem in obtaining accurate exposure information may account in part for the underdiagnosis of FASD because clinicians cannot assume that a woman has a substance-abuse problem without adequate evidence.

### Caregiving Environment

In addition to prenatal alcohol exposure, a child’s caregiving environment also can contribute to problem behaviors that bring children to the attention of clinicians. Many children whose mothers use alcohol in pregnancy have experienced very negative early environments, which may have included parental loss (Berg et al. 2008), institutionalization (in the case of international adoptees [Robert et al. 2009]), abuse and neglect, and frequent foster placements. These environmental factors, in themselves, can lead to significant deficits in cognition and to behavioral and emotional problems and, in extreme cases, may affect physical growth. Although it is possible to discriminate some of these factors from the effects of alcohol exposure in longitudinal studies that follow children over several years, it is extremely difficult to do so in clinical settings. For instance, there currently is no way to discriminate a cognitive deficit associated with prenatal exposure from that resulting from other factors, including environmental under-stimulation. Similarly, hyperactive behavior (or behavioral undercontrol) may be the effect of a teratogen on neurochemistry and anatomy or the consequences of a failure of the caregiving environment to support the child’s development of self-regulation. Thus, although behavior itself may be easy to document if appropriate measures are used, often the cause is less clear, and it can be difficult to argue that prenatal alcohol exposure has produced a particular outcome.

### Comorbidities

A variety of risk factors identified in alcohol-affected individuals may affect later behavior. It is well known that families in which substance abuse is a problem are more frequently characterized by socioeconomic challenges, antisocial behavior, and other drug use (Hussong et al. 2010). Thus, environmental and possibly genetic factors may produce developmental and behavioral outcomes that can co-occur with the effects of prenatal exposure. Given these issues and the caregiving challenges mentioned earlier, it is not surprising that alcohol-affected children often present with a variety of problems, including reactive attachment disorder, conduct disorder, posttraumatic stress disorder, learning disabilities, depression and anxiety, as well as problems in arousal regulation that resemble externalizing behaviors seen in attention deficit hyperactivity disorder (ADHD). At the present time, it is very difficult to establish the extent to which observed disorders can be attributed directly to prenatal exposure; the extent to which they are comorbid, perhaps secondary to the primary effects of alcohol exposure on neurodevelopment (Streissguth and Kanter 1997); or the independent results of other conditions. Regardless of the basis for these disorders, individuals (whether children or adults) who exhibit them will require diagnosis and treatment. Therefore, it is necessary to identify all of the presenting symptoms and to provide a treatment plan that addresses these multiple needs.

### Differential Diagnosis

There are many reasons why it is important to identify the effects of prenatal alcohol exposure on behavior. Most basically, accurate diagnosis allows prevention and treatment efforts to be directed appropriately. For instance, understanding that learning problems result from neurodevelopmental deficits rather than lack of motivation may reframe a child’s learning environment and lead to more targeted and meaningful intervention methods. Over the last decades, clinical and experimental research based on longitudinal studies and animal models have identified a set of outcomes that commonly are seen as a result of prenatal alcohol exposure. Some of these outcomes can be identified reliably and attributed to teratogenic exposure. Other outcomes are observed in clinical settings but may be affected by other prenatal exposures, familial/genetic loading, caregiving environment, and social factors. The following sections review current research findings on the clinical characteristics of alcohol-affected individuals (see Coles 2006; Kodituwakku 2007; Mattson and Riley 1998 for more extensive reviews).

## Neurodevelopmental Effects of Prenatal Alcohol Exposure

### Learning and Memory

Prenatal alcohol exposure is associated with global effects on cognitive functioning, which is ascertained using measures of intelligence (intelligence quotient [IQ]). Early studies suggested that the mean IQ for those diagnosed with FAS was around 70. Thus, half of diagnosed individuals met the criteria for intellectual disability, which is defined as having an IQ score of less than 70 or 2 standard deviations lower than the population mean of 100. People who do not meet the criteria for full FAS generally have scores in the borderline range (i.e., IQs between 70 and 85). However, some physically affected individuals have “normal” IQs (i.e., greater than 85) (Stratton et al. 1996). Scores on other cognitive tests and measures of language usually are consistent with IQ scores. People who demonstrate intellectual disabilities or who function in the borderline range often have difficulties in school and in adapting to everyday life. Those in the borderline range who do not meet the criteria for intellectual disability may not qualify for special educational services or supports, but their functional level can prevent them from benefiting from learning opportunities to the same degree as the average child and often is associated with school failure. These limitations can lead to social and vocational problems, particularly in adolescence and young adulthood when demands for independence increase.

Memory appears to be affected by alcohol to a greater extent than can be explained by general ability (or intelligence). Research on memory in this population has found that there are specific problems in the encoding of information—that is, in the learning of new material or of novel characteristics of information. Using a variety of learning tasks with alcohol-affected children and adults, researchers have identified problems both with simple “rote” memorization and with the employment of strategies to facilitate encoding (e.g. Coles et al. 2010; Kerns et al. 1997; Roebuck-Spencer and Mattson 2004), suggesting effects on both hippocampus and frontal-brain regions. Information that has been encoded effectively (i.e., learned to the level of mastery) appears to be retained as well in this group as in typical individuals but many more trials or different teaching methods may be required to ensure such mastery (Coles et al. 1997; Mattson and Roebuck 2002). These findings, as well as the emerging evidence that white-matter integrity in the brain is compromised in alcohol-affected individuals (Li et al. 2009), suggest that people with FASD may process information more slowly (see Jacobson et al. 1994, discussed below). Therefore, learning time is spent less efficiently and more trials are required to achieve mastery of material. However, research on memory suggests that another important issue for alcohol-affected individuals involves employment of learning strategies. That is, they may not exhibit effective metamemory with the result that they may have more difficulty in selecting and employing effective learning strategies, and may not be aware of the level of effort required to achieve mastery on a particular task.

### Motor and Sensory/Motor Effects

When researchers evaluate study participants in a treatment setting, they detect effects of alcohol exposure on balance and motor control as well as increased clumsiness, abnormal gait, and tremors (Conn-Blower 1991; Marcus 1987; Roebuck et al. 1998; Steinhausen et al. 1993). Such motor problems appear to be the result of teratogenic effects on both central and peripheral nervous systems. Roebuck and colleagues (Roebuck et al. 1998) suggest that damage to the cerebellum from heavy prenatal alcohol exposure may interfere with efficient use of visual and somatosensory system cues, whereas Avaria and colleagues (2004) reported damage to peripheral motor but not sensory neurons.

In the clinic, young children exposed to alcohol often present with early motor problems, including both fine and gross motor delays, which could probably be best described as “mild” in nature compared with the more severe deficits that characterize cerebral palsy and related conditions. These delays often will be observed during infancy and the preschool period and then ignored when the child reaches school age although there often are persistent effects on graphomotor skills (e.g., handwriting) (see figure) and an association with academic problems in certain areas (e.g., math skills).

### Visual/Spatial skills

Visual perception and visual–motor coordination are deficient in alcohol-affected individuals. For instance, in a recent analysis of an international sample, Mattson and colleagues (2010) reported that in addition to executive functioning skills (e.g., planning and organization), spatial processing measures (i.e., spatial recognition and working memory, spatial span, spatial learning, and visual/motor integration) discriminated alcohol-affected individuals from nonaffected control subjects. These findings are consistent with previous studies that suggest specific effects on spatial processing. In a clinical setting, such deficits may be overlooked or misinterpreted. Some signs that there may be problems in this area include difficulties in handwriting, clumsiness, inability to understand gestural communication, and problems in social perception. In addition, because such skill deficits are associated with learning disabilities in mathematics, deficits in these areas may contribute to the frequently observed problems in mathematics reported in this group (Kable et al. 2007).

### Executive Functioning and Effortful Control

#### Arousal and Attention

ADHD is so commonly diagnosed in children with FASD that some researchers have suggested that it is a hallmark of the effects of prenatal alcohol exposure (O’Malley and Nanson 2002). Many children with FAS have difficulty with regulation of arousal and self-regulation, which often may lead to a diagnosis of ADHD (e.g., Nanson and Hiscock 1990; Oesterheld and Wilson 1997) or other alterations in attention (Coles et al. 1997; Streissguth et al. 1986). However, studies of the relationship between FASD and attention have produced inconsistent results. Some studies of sustained attention in longitudinal samples have found deficits in young children, such as increased errors and slower reaction times, but these results have not always been confirmed (see Coles 2006 for a review).

Hypothesizing that early differences in arousal levels seen in alcohol-exposed infants might be associated with later problems in self-regulation and attention, Kable and Coles (2004) examined the relationship between alcohol exposure and information processing in infants. Six-month-old infants who were exposed to alcohol showed differences in several areas when compared with unexposed infants. Alcohol-exposed infants had relative delays in cognitive and motor development and behavior regulation, demonstrated attention differences on a learning task, and were rated as significantly higher in arousal level. Observed differences in information processing speed were consistent with earlier work by Jacobson and colleagues (1994), who found that fixation duration (looking time) was longer in alcohol-exposed infants, implying that alcohol affected the speed of information processing. In other populations, such differences are known to be related to later cognitive functioning. The relationship with arousal regulation in alcohol-exposed children is suggestive of later problems in behavioral regulation and self control. The ability to focus and sustain attention depends on the ability to moderate arousal so that information can be processed efficiently. It may be that alcohol-exposed infants cannot modulate arousal and are therefore less efficient in processing environmental information. Findings in adolescents and adults of inefficiencies in information processing (e.g., Coles et al. 2002; Connor et al.1999) may be influenced by arousal level as well.

#### Executive Function Skills

Executive functioning involves higher-order cognitive processes that include attentional regulation, working-memory skills, planning and organizational thinking, and problem solving (Morris 1996). Both clinical descriptions of children with FAS and longitudinal exposure studies suggest that prenatal alcohol exposure consistently affects executive functioning. A number of different cognitive elements, including metamemory and active working memory usually are understood to be part of executive functioning. Active working memory involves mental manipulation of elements stored briefly in short-term memory and has been referred to as a “mental scratch pad” (Ashcraft 1995). It requires that information be stored and processed in meaningful “units” at a given moment in time. In alcohol-affected individuals, problems in this aspect of cognition have been identified as early as 3 months of age (Jacobson et al. 1993), when alcohol-exposed infants had more difficulty than non-exposed control subjects in maintaining and manipulating three items of information simultaneously. Infants also were found to have difficulty shifting from one rule to another (i.e., learning to anticipate where items would be presented next) and with mastering complex spatial sequences. Kodituwakku and colleagues (1995) used several neurodevelopmental tasks tapping active working memory with school-aged children and adolescents and argued that working memory problems were the basis for much of the cognitive impairment seen in FAS. In practical terms, such a deficit influences daily functioning and academic performance. For instance, children diagnosed with FAS consistently show impairments in mathematics and assessment of math on intelligence tests relies heavily on active working-memory skills by requiring the manipulation of numbers and arithmetic operations in short-term memory.

People with FASD also have impairments in planning, organization, and problem-solving aspects of executive functioning. For example, problems with planning have been demonstrated on progressive planning tasks and reversal shift tasks in which an individual is asked to change an established response when presented with different contingencies. See the textbox for descriptions of these and other experimental tasks mentioned in this article. Both kinds of tasks originally were used to measure the effects of frontal-lobe damage in adults (Shallice 1982). Using both kinds of tasks, children with FAS have been found to perform more poorly than control subjects and to make perseveration errors (i.e., repetition of responses that are incorrect or not reinforced) (Coles et al. 1997; Kerns et al. 1997; Kodituwakku et al. 1995), suggesting that they have difficulty incorporating environmental feedback to correct a response. Children with FAS typically have difficulty shifting attention from one stimulus to another even when it is the appropriate response. This is particularly true for what are called nonreversal shifts (see textbox), (Coles et al. 1997; Kerns et al. 1997; Kodituwakku et al. 1995), a response that implies difficulties with inhibition of learned responses or an inattentiveness to new information. Kodituwakku and colleagues (2001) also found that adding emotional elements to these cognitive tasks further decreased performance by those with FASD, perhaps because problems in regulation of emotional arousal affected performance.

## Discriminating Alcohol Effects From Other Developmental Disorders

Some of the behavioral and learning characteristics seen in FASD are similar to those observed as a result of other conditions. Outcomes frequently associated with FASD include developmental and learning problems, academic failure, particularly in the area of mathematics, and disorders of regulation and conduct. Determining whether observed behaviors are associated with FASD or other conditions can be both challenging and rewarding. The relationship between FASD and ADHD provides an example.

### FASD and ADHD

It is estimated that more than 70 percent (Burd et al. 2003; Dalen et al. 2009) of alcohol-affected children presenting for treatment receive a diagnosis of attention deficit disorder, usually ADHD. However, investigation of the overlap between FASD and ADHD suggests that some caution should be exercised in making this diagnosis. Despite its variability, development tends to flow into certain “final common pathways,” resulting in similar patterns of developmental delay and externalizing disorders. Thus, given the current methods for the diagnosis of attention deficit disorders (i.e., questionnaires and interviews), it is not surprising that ADHD is diagnosed frequently in alcohol-exposed children as it is in many other conditions, including autism, genetic disorders, and the effects of trauma. However, when methods are used that allow a more critical examination of cognitive and behavioral characteristics, it is possible to discriminate FASD from ADHD. Coles and colleagues (1997) directly compared prenatally exposed school-aged children to those diagnosed with ADHD and reported that it was possible to discriminate these groups based on both the standard parent and teacher questionnaires that usually are used in ADHD diagnosis and on neurocognitive tests. More specifically, alcohol-exposed children had fewer behavior problems than children with ADHD with similar IQ levels who were recruited from clinical services. In addition, they were more likely to show deficits in encoding of information and in the ability to effectively “switch” their attention when it was required. In contrast, the children with ADHD, who were drawn from the same low-income, minority population, had more externalizing disorders and greater problems in focusing and sustaining attention. More recently, several studies have directly compared the characteristics of children with FASD and ADHD. Kooistra and colleagues (2009) specifically examined sustained attention and arousal modulation in these groups using a continuous performance task, which measures sustained attention; and the Go-No-Go protocol, which measures the ability to inhibit a response, something that is difficult in ADHD (see textbox). The researchers found a good deal of variability in the response of these clinical groups as well as decrements in performance relative to control subjects under most conditions. They attributed some of the observed performance differences to alterations in the ability to modulate arousal adaptively, both in the FASD and ADHD groups. Vaurio and colleagues (2008) compared executive functioning in children with prenatal alcohol exposure and ADHD and found that alcohol exposure was associated with more extensive deficits than was ADHD, although there were significant differences in IQ between the groups that may have affected performance. Coffin and colleagues (2005) used an eyeblink reflex response paradigm (see textbox) to compare children with FASD, ADHD, and dyslexia with nonaffected control children. This study is notable in that all children had IQs in the average range without the usual IQ discrepancy between FASD and comparison groups. Results indicated that FASD and dyslexia were associated with impaired learning of this cerebellum-based reflexive response, but ADHD was not. In a study examining motor balance and motor problems in FASD and ADHD, Kooistra and colleagues (2010) found that although both clinical groups showed impairments relative to control subjects, the ADHD group showed more extensive impairment. The FASD group’s performance was more likely to be normal. Burden and colleagues (2010) used a longitudinal sample to compare adolescents with prenatal alcohol exposure and ADHD with peers diagnosed with ADHD who were not exposed to alcohol. Using a Go-No-Go paradigm, they concluded that these groups could be discriminated on the basis of measures of brain activity (i.e., event-related potentials) and argued that these groups have unique neurophysiological profiles.

**Experimental Tasks Used to Study the Effects of Prenatal Alcohol Exposure**

Progressive Planning Tasks are used to evaluate the ability to plan a series of actions. In the test used by Kodituwakku and colleagues (1995), children are presented with a 3-peg board with movable colored beads. The children are given a picture showing how the beads should look after being rearranged and asked to create and implement a plan to get the beads into the right configuration. The children are also given rules to follow that allow the rearrangement to be successful only when a specific plan is followed. These rules are 1) only one bead can be moved at a time, and 2) once removed from its initial position, a bead must not be returned to that position. The person doing the test has to remember and follow the rules while they are making and implementing their plan. They also have to “think ahead” several steps to envision how the plan will work out in practice.

**Reversal/shift procedures** have been used to demonstrate both the development of cognition and the effects of specific brain damage (Briar and Jacobs 1972). In these procedures (including the Wisconsin Card Sorting Test [Heaton 1999], described below), the person being tested is presented with an array (2 to 4) of symbols and rewarded for choosing the correct one. Each symbol has several characteristics that can be used to discriminate the correct choice, usually color, number, and shape. For example, one card might show a red circle (i.e., having the dimensions red, 1, and circle) while another might show five green stars (having the dimensions, green, 5, and star). After several “trial and error” attempts, during which the same dimension (e.g., red) is always correct, the person learns that this is the right choice. After the choice is made correctly for a number of trials (usually 9 of 10), the correct response is “shifted.” If it is a “reversal” shift, then the change is along the same dimension, from “red” to “green” as the correct choice. If it is a “nonreversal” shift, then a different dimension is used so that the new correct choice might be “circle.” Usually, the nonreversal shifts are more difficult than the reversal shifts. Young children and individuals with damage in the frontal regions of the brain have difficulty with these shifts and often “perseverate” on the original choice without being able to “shift.” This pattern was noted in children with FASD by Coles and colleagues (1997).

**A continuous performance task** measures sustained and selective attention and impulsive responding. Using a computer, the person being tested is presented with a series of symbols, usually letters, and is asked to respond when they see or hear certain symbols or series of symbols. The simplest of these tests are “vigilance” tasks or measures of the ability to sustain attention on a task. In such tests, a series of different letters may be presented one at a time and the person being tested is required to respond (usually by pressing a button) when a particular symbol appears. The symbol is presented in a random sequence with a number of other similar symbols so that the person being tested has to maintain their attention on the task to avoid missing the target. If the target is missed, they are said to have made an “omission” error. If they respond to targets other than the correct target, they are said to have made a “commission” error. More demanding versions of the test require the person taking the test to watch for a pattern of symbols (e.g., an “X” that follows an “A”). This is the only correct response. Therefore, if they see an “X” that was not preceded by an “A”, they must inhibit their tendency to respond. This task requires both vigilance (attention) and executive control of impulsive responding.

**Go-No-Go** tests are used to measure the capacity for sustained attention and control of responses. Generally the person being tested is presented with two categories of stimuli (e.g., red/green; “X” versus other letters) and required to perform an action (e.g., pressing a button) when presented with one stimulus and to inhibit their response (not press the button) when presented with the other stimulus. In contrast with the continuous performance task, the person being tested is required to perform the response for most stimuli and to inhibit the response only when presented with the target stimulus. They are asked to respond as quickly as possible without sacrificing accuracy and response time (Burden et al. 2010).

**Eyeblink reflex response paradigm or eyeblink conditioning** is designed to study the aspects of learning and memory that depend on the function of the cerebellum. This method can be used in both human and animal studies and uses a form of classical conditioning in which a neutral (conditioned) stimulus, usually a sound, is paired with another (unconditioned) stimulus. This second stimulus elicits some kind of physiological response. In the case of the eyeblink conditioning, the unconditioned stimulus is a puff of air directed at the eye, which causes a reflexive eyelid blink. This response occurs without any previous training and is therefore called an “unconditioned” response. When a sound is repeatedly paired with the puff of air, the eyeblink comes to be elicited by the sound as well as the air puff and, eventually, the sound itself will produce the eyeblink even if the air puff is not presented. It is then called a “conditioned response.” This process of association is called classical conditioning.

ReferencesBurdenMJJacobsonJLAn event-related potential study of response inhibition in ADHD with and without prenatal alcohol exposureAlcoholism: Clinical and Experimental Research34461762720102010256810.1111/j.1530-0277.2009.01130.xBriarNJacobsPIReversal shifting: Its stability and relation to intelligence at two developmental levelsChild Development434123012411972ColesCDPlatzmanKARaskind-HoodCLA comparison of children affected by prenatal alcohol exposure and attention deficit, hyperactivity disorderAlcoholism: Clinical and Experimental Research21115016119979046388HeatonRKWisconsin Card Sorting Test: Computer Version 3 for Windows: Research EditionLutz, FLPsychological Assessment Resource1999KodituwakkuPWHandmakerNSCutlerSKSpecific impairments in self-regulation in children exposed to alcohol prenatallyAlcoholism: Clinical and Experimental Research196115815641995874982710.1111/j.1530-0277.1995.tb01024.x

Thus, it appears that both similarities and distinctions can be identified in the behavioral, cognitive, and motor functions that characterize these different diagnostic groups. For that reason, appropriate evaluation could improve diagnosis and allow for more effective and sensitive treatment than is probably occurring in many settings at the present time. For instance, although stimulants are widely prescribed to children with FASD, in the few medications studies that have been reported, results suggest that stimulant medications that are usually effective for the attention deficit subtypes are not helpful for FASD (Frankel et al. 2006; Oesterheld et al. 1998; Snyder et al. 1997). Similarly, if one of the underlying problems with ADHD is impulse control, and children with FASD have difficulty in encoding information and require more time to learn, focusing on inhibiting impulsive responding may not be a useful strategy for the alcohol-affected group.

## Summary and Conclusions

For a number of reasons, FAS and other conditions associated with prenatal alcohol exposure are probably underdiagnosed in clinical settings that focus on behavior and developmental problems. In addition to the factors reviewed in this article, the absence of an alcohol-related diagnostic category that can be used by behavioral health practitioners contributes to this oversight. However, attributing the effects of the neurodevelopmental impact of alcohol exposure to other factors (like ADHD or behavioral disorders) does a disservice to the individuals with FASD and to their families. As has been demonstrated by recent research, appropriate identification of the behavior and developmental problems associated with prenatal exposure will allow more targeted and effective interventions. Understanding the deficits in cognition that produce the behaviors that are observed clinically will improve outcomes for affected individuals and allow more effective use of resources. For these reasons, it is important to identify factors that discriminate FASD from other clinical conditions and make this information widely available. Previous research, reviewed here, has identified a number of patterns in behavior and cognition that appear to be associated with FASD. The next important step is to examine the degree to which these patterns are distinct to FASD and how they can be discriminated from other developmental and behavioral disorders. When this research has been carried out, the necessary tools will be available to allow accurate diagnoses in clinical settings to become the norm rather than the exception.

## Figures and Tables

**Figure:**
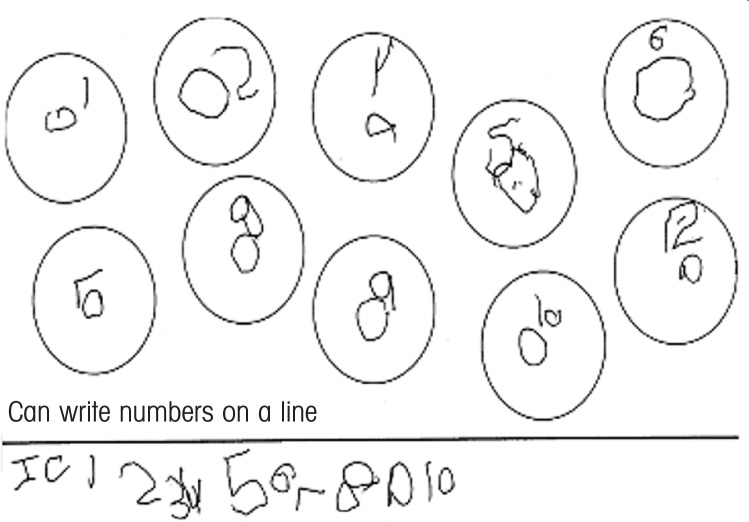
Handwriting sample from a 6-year-old affected by prenatal alcohol exposure. The child was instructed to write each of the numbers from 1 to 10 in a circle and to write the numbers on the line.
